# Hormonal signaling in glioblastoma: emerging roles in stemness and tumor plasticity

**DOI:** 10.3389/fendo.2026.1924678

**Published:** 2026-10-05

**Authors:** Jessica García-Sánchez, Alicia Cañas-Linares, Patricia Garcia-Lopez

**Affiliations:** Laboratory of Pharmaco-Oncology and Nanomedicine, Basic Research Division, National Cancer Institute, Mexico City, Mexico

**Keywords:** glioblastoma, glioma stem cells, hormonal signaling, plasticity, resistance, stemness, temozolomide

## Abstract

Glioblastoma (GBM) is characterized by pronounced cell-state plasticity and functional heterogeneity, which drive therapeutic resistance, tumor recurrence, and disease evolution. Although these processes have traditionally been viewed through a predominantly genomic perspective, emerging evidence identifies endocrine signaling as a critical regulator of glioblastoma cell states. In this review, we propose that hormones function not as independent signaling pathways or merely as promoters of tumor growth, but as systemic regulators that integrate physiological and microenvironmental signals to modulate transitions among stem-like, proliferative, invasive, and treatment-resistant cellular states. We analyzed the current evidence on the roles of sex steroids, glucocorticoids, the IGF/insulin axis, melatonin, thyroid hormones, and prolactin, highlighting how these endocrine signals converge on common molecular nodes, including PI3K/AKT/mTOR, MAPK/ERK, JAK/STAT3, and epigenetic regulators such as EZH2, to coordinate stemness-associated programs, adaptive cell-state responses, and therapeutic adaptation. We also discussed the dual nature of various hormonal axes, including differential activity of estrogen receptor isoforms, the role of the androgen receptor in maintaining self-renewal, the impact of glucocorticoids on adaptive cellular reprogramming, and the ability of melatonin to destabilize stem-like cellular states. Finally, we discuss how sex-based differences in GBM incidence and outcome -driven by both biological and non-biological factors- influence diagnosis, access to treatment, and current interpretations of the global epidemiology of glioblastoma. Collectively, we propose a conceptual framework that redefines GBM as an endocrine-modulated disease, in which hormonal signaling may modulate tumor cell state adaptation. This perspective provides a basis for developing therapeutic strategies that target shared molecular convergence nodes while integrating the biological complexity of GBM with its broader clinical and socioeconomic context.

## 1 Introduction

Glioblastoma (GBM) is the most common and aggressive primary malignant tumor of the central nervous system (CNS) in adults, with an estimated global incidence of 3–5 cases per 100,000 individuals annually (1, 2). Despite advances in neurosurgery, radiotherapy, and chemotherapy, the current standard of care -based on maximal safe surgical resection followed by concomitant radiotherapy with temozolomide chemotherapy (3)-, continues to provide only modest clinical benefit. Likewise, complementary therapies such as Tumor Treating Fields (TTFields), which deliver low-intensity alternating electric fields, and the monoclonal antibody bevacizumab have improved selected clinical outcomes without substantially extending overall survival. Consequently, median overall survival remains approximately 15 months, fewer than 5% of patients survive beyond five years, and tumor recurrence is virtually inevitable (1). Limited response is a major resistance mechanism. The cytotoxic effect of temozolomide results from the alkylation of guanine at the O6 and N7 positions of DNA. The DNA repair enzyme O6-methylguanine-methyltransferase (MGMT) removes these adducts, leading to resistance and recurrence. Other therapeutic resistance mechanisms in GBM include overexpression of ATP-binding cassette (ABC) transporters, which promote temozolomide efflux at the cellular and blood-brain barrier levels. Additional resistance mechanisms include intratumoral cellular heterogeneity, adaptive transcriptional and epigenetic reprogramming, the presence of cancer stem cells (CSC), and the persistence or reacquisition of stem-like states after treatment (4). This adaptive behavior is now recognized as a defining biological hallmark of GBM and arises from its pronounced intratumoral heterogeneity. Rather than a homogeneous cell population, GBM comprises multiple coexisting cellular states that continuously evolve in response to genetic and epigenetic alterations, tumor microenvironmental interactions, hypoxia, metabolic and endocrine signals, and mechanisms of stemness and cellular plasticity. This dynamic organization promotes the selection of therapy-resistant populations and largely explains the high invasiveness, therapeutic resistance, and inevitable recurrence that characterize this malignancy.

Among the mechanisms driving this heterogeneity, stemness and cellular plasticity have emerged as closely interconnected biological processes. Stemness is a functional state that endows specific cells with self-renewal capacity and differentiation potential (5), whereas cellular plasticity is the ability of tumor cells to transition between distinct phenotypic states in response to intrinsic and extrinsic stimuli, including therapy-induced stress (6, 7). Within this framework, stemness is no longer an exclusive property of a stable population of cancer stem cells but a dynamic, reversible cellular state that can be acquired or lost through plastic phenotypic transitions (8). This perspective redefines the classical hierarchical model of GBM and provides a biological explanation for the tumor’s remarkable ability to regenerate after treatment.

Interconnected transcriptional, epigenetic, and metabolic programs orchestrate these phenotypic transitions by regulating essential processes, including cell survival, DNA damage repair, energy metabolism, and stress responses (9). Importantly, these regulatory networks do not operate in isolation. Instead, components of the tumor microenvironment, including hypoxia, extracellular matrix architecture (10, 11), infiltrating immune cells, and endocrine signals, continuously reshape tumor cell functional states.

Several studies show that hormonal signaling modulates GBM phenotypes relevant to stemness, survival, metabolism, and treatment adaptation. Hormones such as estrogens, progesterone, androgens, glucocorticoids, IGF/insulin, and thyroid hormones regulate molecular programs involved in proliferation, survival, metabolism, and stemness maintenance. Rather than acting independently, these endocrine pathways converge on shared regulatory networks that promote tumor adaptation and therapeutic resistance (12–14). This emerging concept positions endocrine signaling as an integral component of the tumor ecosystem, extending beyond its traditional role as a systemic physiological regulator.

Understanding how endocrine signals coordinate stemness, cell-state plasticity, and the tumor microenvironment may reveal previously unrecognized biological vulnerabilities in GBM. From a therapeutic perspective, this knowledge supports the development of combinatorial strategies that target convergent signaling hubs, including the AKT/mTOR and STAT3 pathways, where multiple redundant signals sustain the aggressive tumor phenotype. Within this conceptual model, the endocrine system should no longer be regarded as an accessory regulator but as a central component of glioblastoma biology and a promising therapeutic target.

### 1.1 Glioma stem cells: the dynamic core of cell-state plasticity

Glioma stem cells (GSCs) were originally conceptualized as a tumor subpopulation defined by self-renewal, multipotency, and tumorigenicity. This hierarchical model proposed a relatively stable cellular compartment responsible for tumor propagation and therapeutic resistance. However, evidence accumulated over the past decade has substantially transformed this view, showing that stem-like properties do not constitute a fixed cellular identity but rather a dynamic, reversible state within a continuum of functional cell states (5, 15).

In this emerging context, previously differentiated cells can acquire a stem-like identity in response to signals from the tumor microenvironment, metabolic changes, or therapeutic pressure. This phenotypic reprogramming has been extensively documented in both experimental and clinical settings, supporting the role of therapy-induced phenotypic reprogramming in GBM progression and recurrence (16–18). Consequently, GBM’s remarkable regenerative capacity after therapy does not depend exclusively on the persistence of a pre-existing stem cell population but reflects the tumor ecosystem’s ability to continuously reconfigure its cellular states.

At the molecular level, therapy-induced state reprogramming is orchestrated by highly interconnected transcriptional, epigenetic, and metabolic networks that enable reversible transitions among proliferative, quiescent, invasive, and treatment-resistant states (15, 17). Rather than following linear developmental trajectories, these transitions occur within a dynamic cellular landscape in which stemness emerges as transient configurations favored by specific microenvironmental conditions. Accordingly, stemness in GBM is no longer viewed as an intrinsic property of a discrete cell population but as an emergent property of the tumor ecosystem.

Multiple extrinsic signals modulate the dynamics of these cellular states. Among them, endocrine signaling has emerged as a particularly important systemic regulator, capable of integrating physiological and microenvironmental information to influence both the stability of and transitions between stem-like states. This perspective positions hormones not merely as isolated mitogenic stimuli, but as integrative regulators of the molecular networks involved in adaptive cell-state regulation and tumor evolution.

### 1.2 Glioblastoma: a hormone-sensitive tumor

The central nervous system (CNS) can synthesize steroid hormones from cholesterol, producing neurosteroids that actively regulate cellular physiology and the neural microenvironment (19). Although GBM is not considered a classically hormone-dependent tumor, as are breast or prostate cancers, glioma cells express a broad repertoire of hormone receptors, with expression that is highly heterogeneous both among patients and within individual tumors. Accordingly, GBM is more appropriately defined as a hormone-sensitive tumor, rather than a strictly hormone-dependent malignancy. Within this framework, hormonal signals are not considered primary oncogenic drivers; however, they can significantly modulate key biological processes, including proliferation, invasion, survival, and the acquisition of stem-like phenotypes (20, 21).

Hormone receptors belong to a superfamily of ligand-activated transcription factors that regulate gene expression during oncogenesis and CNS tumor progression (20). Several studies have shown that estrogens, progesterone, glucocorticoids (including dexamethasone and cortisol), and thyroid hormones (T3/T4) each influence the aggressiveness of glioblastoma. Nevertheless, researchers have traditionally studied these endocrine pathways in isolation, with limited attention to how they integrate into glioma stem cell biology and the dynamic regulation of stemness-associated and adaptive phenotypes.

More recently, accumulating evidence has shown that hormonal signaling can modulate glioma stemness-associated phenotypes and treatment-adaptive responses in GBM. These pathways help stabilize undifferentiated cellular states and promote self-renewal, thereby sustaining resistance to chemotherapy and radiotherapy. Thus, hormonal signaling appears to regulate not only tumor proliferation but also the dynamic architecture of cell-state transitions that ultimately drive tumor evolution. From a translational perspective, this emerging framework has stimulated growing interest in therapeutic strategies targeting hormone receptors, including receptor antagonists, selective receptor modulators, and combination therapies with chemotherapy or immunotherapy. Nevertheless, despite the substantial preclinical evidence, clinical validation of these approaches remains limited (12, 13).

## 2 Sex hormones as major modulators of GBM stemness

### 2.1 Estrogenic activity in the modulation of GSCs

Estrogen signaling in GBM has complex effects and must be interpreted by receptor class and specific isoform. For estrogen receptor alpha (ERα), the canonical form is typically the full-length ERα66 isoform, whereas ERα46 and ERα36 are shorter variants with distinct domain compositions and signaling properties. Estrogen receptor beta (ERβ) likewise comprises functionally non-equivalent isoforms, including ERβ1 and ERβ5, while GPER is a G protein-coupled estrogen receptor that mediates rapid, non-genomic signaling. The literature on GBM provides the strongest evidence for ERα36, ERβ1, and ERβ5 isoforms, as well as GPER-mediated signaling; therefore, effects attributed to a receptor variant should not be generalized to the entire ERα or ERβ receptor family.

Depending on the isoform, these findings challenge the traditional paradigm that assigns an exclusively pro-tumorigenic role to ERα and predominantly tumor-suppressive functions to ERβ. Several studies indicate that the biological response to estrogens is largely determined by differential expression of receptor isoforms, rather than by receptor subtype alone. In GBM, ERα36 and ERβ5 variants have emerged as important mediators of tumor progression, chemoresistance, and mitogenic signaling. ERα36, for example, promotes these effects through functional crosstalk and co-localization with the epidermal growth factor receptor (EGFR), thereby amplifying signaling programs associated with tumor aggressiveness (22–24). In contrast, the ERβ1 isoform exerts a markedly tumor-suppressive role by antagonizing key inflammatory and self-renewal pathways, including NF-κB and JAK/STAT3 signaling (25). Through this mechanism, ERβ1 restrains the transition toward mesenchymal-like cellular states, which are closely associated with invasion, therapeutic resistance, and poor clinical outcome (26). These opposing functions, driven by splicing events that alter the recruitment of transcriptional coregulators, suggest that estrogen signaling extends well beyond its classical role in regulating cell proliferation. Instead, it functions as a dynamic regulator of the transcriptional and epigenetic landscape that governs proliferative, migratory/invasive, and balance stemness-associated programs.

Although studies specifically focused on GSC remain relatively limited, available functional evidence strongly supports this model. Sareddy et al. (27) demonstrated that ERβ activation with the agonists LY500307 and liquiritigenin markedly reduces neurosphere formation, impairs self-renewal capacity, and decreases expression of stemness-associated markers, including SOX2, CD133, and SSEA-1 in patient-derived GSCs. Moreover, pharmacological activation of ERβ significantly reduced tumor growth and prolonged survival in intracranial orthotopic mouse models, confirming that this pathway restricts stemness-associated programs.

Complementing these genomic effects, non-genomic estrogen signaling also helps regulate GSC properties. Méndez-Luna et al. (28) showed that simultaneous inhibition of the G protein-coupled estrogen receptor (GPER) and the anti-apoptotic protein Bcl-2 using tetrahydroquinoline derivatives significantly reduces neurosphere formation and suppresses GSC proliferation and migration in three-dimensional culture models. These findings indicate that estrogen signaling regulates not only tumor proliferation but also the molecular programs governing glioblastoma cell-state transitions. Rather than acting simply as mitogenic stimuli, estrogen receptors function as context-dependent determinants of stem-like identity, shaping the dynamic equilibrium between differentiation and stemness. This emerging framework positions the estrogen axis as a context-dependent modulator of GSC-associated phenotypes and highlights its therapeutic potential for disrupting the adaptive transitions that drive glioblastoma progression.

### 2.2 Progesterone signaling: a modulator of stemness-associated phenotypes in glioblastoma

Progesterone signaling has been extensively implicated in GBM biology, although direct evidence that progesterone regulates cell-state plasticity remains limited. In tumor tissues and cell lines expressing the progesterone receptor (PR), progesterone exposure has been shown to promote tumor growth, proliferation, and migratory capacity, both *in vitro* and *in vivo* (29, 30). Furthermore, progesterone promotes infiltration and cell motility by activating PR-dependent classical nuclear signaling and rapid non-genomic pathways linked to actin cytoskeleton remodeling, which in turn activate various kinase cascades (31, 32).

Beyond proliferation, progesterone signaling appears to play an important role in acquiring adaptive and chemoresistant phenotypes. Transcriptomic and gene expression analyses show that progesterone significantly remodels the tumor microRNA (miRNA) landscape, reducing expression of tumor-suppressor miRNAs such as miR-135b and miR-29b. These changes affect cellular programs related to cell-cycle progression, apoptosis evasion, and stress adaptation (33). However, the biological response depends on concentration. While physiological concentrations favor tumor progression, high pharmacological doses reduce glycolytic metabolism and induce premature senescence by inhibiting the PI3K/Akt/mTOR axis (34, 35). This metabolic response creates selective pressure that drives the tumor to activate compensatory mechanisms, increasing phenotypic heterogeneity and promoting adaptation.

Several studies have reported the effects of progesterone on GSC growth and differentiation-associated features in gliomasphere models. In these systems, hormonal treatment significantly increases the number and size of clonogenic gliomaspheres derived from human cell lines (32), supporting the notion that progesterone promotes gliomasphere growth and expands stem-like cellular states. Local expression of progesterone-induced blocking factor (PIBF), a paracrine mediator that promotes immunotolerance in the tumor microenvironment and helps maintain cellular heterogeneity, amplifies this response (31).

These findings suggest that progesterone signaling extends beyond its classical role in regulating proliferation and invasion and functions as an important orchestrator of adaptive cell-state transitions. Although the underlying mechanisms in GSC populations remain unclear, current evidence supports that progesterone signaling modulates proliferation, invasion, metabolism, stemness-associated features, and treatment response, thereby contributing to glioblastoma evolution.

### 2.3 Androgen activity in the maintenance of GSC

Several studies have documented androgen receptor (AR) overexpression in gliomas, particularly in glioblastoma stem cells (GSCs). Both transcriptional and protein AR expression increase progressively with histological grade and tumor malignancy (36, 37). Furthermore, detection of functional AR in multiple GBM cell lines, including U87MG, T98G, and LN229, confirms its role as a key mediator in the progression of this neoplasm (38). Functionally, AR-mediated signaling directly helps maintain the stem-like phenotype in glioblastoma. Although this receptor is not restricted to the CD133+ subpopulation and does not serve as a lineage marker for GSCs, its pharmacological inhibition with the antiandrogen enzalutamide significantly reduces the fraction of CD133+ cells, decreases neurosphere-forming capacity, and attenuates the frequency of cells with tumor-initiating capacity, both *in vitro* and in orthotopic murine models (39). At the molecular level, enzalutamide reduces expression of the essential transcription factors NANOG, OCT4, and SOX2, which are key regulators of pluripotency and self-renewal programs (39). Taken together, these findings support the idea that the AR acts as a functional modulator of adaptive stemness, regulating the dynamics of cell states.

Further evidence indicates that the androgen axis also actively contributes to therapeutic resistance. Clinically approved or evaluated second-generation antiandrogens, such as enzalutamide and seviteronel, slow tumor growth and increase the radiosensitivity of cell lines and AR-positive patient-derived xenografts by interfering with repair mechanisms for radiation-induced damage (40). Additionally, Díaz Méndez et al. (41) showed that AR silencing, either through a tumor-suppressor microRNA damage signature (miR-1/miR-26a-1/miR-487b) or through pharmacological blockade with enzalutamide, decreases neurosphere formation by repressing WT1 and FOXA1 and sensitizing GSCs to cytotoxic treatment with temozolomide.

Additional modulators of androgen signaling also influence stemness-associated phenotypes, proliferation, and cell survival. Finasteride, a 5α-reductase inhibitor that crosses the blood-brain barrier, compromises GSC maintenance and reduces tumor proliferation by lowering intracellular reactive oxygen species (ROS). This, in turn, inhibits the β-catenin pathway and destabilizes pluripotency-associated programs (42). Similarly, androgen receptor (AR) antagonists such as apalutamide (RNA-509) and bicalutamide induce concentration-dependent apoptosis in both conventional cell lines and glioma-initiating cells (43).

These findings position androgen signaling as a central regulator of stemness maintenance and therapeutic adaptation in glioblastoma. Rather than acting solely as a driver of tumor proliferation, the androgen axis appears to modulate stemness-associated features and therapeutic response. This perspective also provides a biological basis for exploring the higher incidence of GBM in men, who, under physiological conditions, have higher circulating and tissue androgen concentrations (39, 44).

## 3 Metabolic, stress-related, and circadian rhythm hormones

### 3.1 IGF/insulin axis and stem cell maintenance

Dysregulation of receptor tyrosine kinase (RTK) signaling is a hallmark of GBM biology, particularly in stem-like cellular subpopulations that rely on self-renewal, stem-like maintenance, and metabolic adaptation to persist (45–47). Among these pathways, activation of the IGF/insulin axis and its downstream effectors converge on the PI3K/AKT/mTOR signaling cascade, which integrates extracellular matrix remodeling and metabolic reprogramming (48–51).

Several studies have demonstrated that insulin-like growth factor receptor 1 (IGF-1R) signaling is a critical mediator of adaptive radioresistance in GSCs (52). The magnitude of this response depends on both cellular identity and the oncogenic context within the glioma development hierarchy (53). Activation of the IGF/insulin axis enables both glioma-initiating cells and quiescent tumor populations to enter transient adaptive states that promote survival after cytotoxic therapy and are associated with poor clinical outcomes (54–57). These adaptive programs are closely coupled to molecular networks that regulate proliferation, maintenance of the undifferentiated state, and the metabolic reprogramming required to sustain elevated biosynthetic demand (58, 59). STAT3 (signal transducer and activator of transcription 3) is the central node integrating these processes. Upon phosphorylation, STAT3 translocates to the nucleus, where it functions as a master transcriptional regulator of gene programs associated with pluripotency, cell survival, and adaptation to hypoxic and genotoxic stress (60, 61). Accordingly, STAT3 links metabolic signaling, stemness, and therapeutic resistance by integrating signals from both the tumor microenvironment and systemic endocrine pathways (62–66).

The biological relevance of this signaling network is further supported by several experimental therapeutic strategies. Radiation induces the release of extracellular vesicles enriched for miR-603, promoting the acquisition of an IGF1-dependent stem-like state within the tumor microenvironment (67). Conversely, targeted delivery of liposome-encapsulated miR-603 to patient-derived GSCs effectively reverses this adaptive response and restores radiosensitivity (68). Likewise, dimethylaminomicheliolide (DMAMCL) suppresses glioma cell proliferation by inducing reactive oxygen species (ROS) accumulation and autophagosome formation, while simultaneously inhibiting Akt/mTOR signaling, thereby disrupting metabolic programs that sustain stem-like phenotypes (69).

These findings indicate that the IGF/insulin axis extends well beyond its classical role in metabolic regulation. From this perspective, the convergence of metabolic signaling and the networks that control cell identity favors drug-tolerant subpopulations, thereby contributing decisively to tumor heterogeneity and the adaptive evolution of GBM.

### 3.2 Glucocorticoid signaling in GBM: implications for adaptive reprogramming and treatment response

GBM causes progressive neurological dysfunction through diffuse infiltration of the brain parenchyma and the development of peritumoral edema. Glucocorticoids play a dual role in GBM, modulating the tumor microenvironment to reduce edema or inducing chemoresistance mechanisms that promote tumor progression. Dexamethasone (DEX) is a synthetic glucocorticoid widely used in patients with brain tumors and remains part of standard therapy for controlling cerebral edema because of its potent anti-inflammatory effects (70). However, a recent meta-analysis reported a statistically significant association between DEX administration and reduced overall and progression-free survival in patients with GBM (70). These findings have prompted growing interest in understanding how glucocorticoids influence tumor subpopulations with stem-like properties and high adaptive capacity.

Experimental studies using patient-derived primary GBM cultures have shown that DEX exposure enhances neurosphere-forming capacity and increases the expression of stemness-associated markers, including CD133, thereby promoting the acquisition of a stem-like phenotype and conferring resistance to temozolomide (71).

These findings suggest that glucocorticoid receptor (GR) activation not only modulates the inflammatory response but also promotes the functional reprogramming of tumor cells toward more treatment-resistant states. Supporting this concept, transcriptomic analysis has identified a GR-dependent gene signature comprising more than 30 genes whose expression correlates with poor patient survival (72). This transcriptional program preferentially activates mesenchymal differentiation and DEX-responsive transcriptional programs associated with CEBPB (CCAAT/enhancer-binding protein beta) activation, with particularly pronounced effects in quiescent GSC populations (72). Available evidence indicates that GR signaling can promote treatment-resistant transcriptional reprogramming in GSC models rather than merely mediating symptomatic edema control. By promoting a stem-like transition, reinforcing mesenchymal programs, and enhancing resistance to chemotherapy, glucocorticoid signaling contributes to tumor adaptation and disease progression. These findings underscore the need to carefully balance the undeniable clinical benefits of DEX against its potential protumorigenic effects and highlight the importance of developing therapeutic strategies that preserve its anti-edematous efficacy while minimizing activation of molecular programs associated with stemness.

### 3.3 Melatonin: regulation of GSC maintenance and therapeutic response in GBM

Melatonin combines systemic circadian and receptor-mediated actions, and exerts potent antitumor effects on GSCs, reducing proliferation, clonogenic capacity, and tumor sphere formation *in vitro*, while impairing tumor-initiating capacity in orthotopic patient-derived xenograft models (73). These functional effects are accompanied by downregulation of stemness-associated markers, including SOX2, OCT4, NANOG, and CD133, indicating a progressive loss of the stem-like phenotype after melatonin exposure (73, 74).

At the molecular level, melatonin suppresses core self-renewal pathways, particularly the AKT–EZH2–STAT3 and EZH2–NOTCH1 signaling axes. Specifically, melatonin reduces EZH2 (Enhancer of Zeste Homolog 2) phosphorylation and activity, thereby disrupting the transcriptional and epigenetic programs required for GSC maintenance (73, 74). Many of these effects are mediated by the transmembrane melatonin receptors MT1 and MT2. In high-grade gliomas, these receptors are dysregulated, with reduced MT1 and increased MT2 levels compared with non-neoplastic brain tissue (75).

Pharmacological manipulation of melatonin receptors further supports receptor-specific effects in GBM models. Luzindole, a non-selective MT1/MT2 melatonin receptor antagonist with higher affinity for MT2 (76), modulates cellular pathways involved in glioma progression. Therefore, findings with this compound could reflect MT2-preferential antagonism in the experimental context, inhibiting signaling pathways that regulate cell proliferation and glioma development (77). Other compounds, such as DH97, have been used as more MT2-selective pharmacological tools in receptor studies. These findings support the conclusion that selective MT2 receptor antagonists, using DH97, exert antitumor effects *in vitro* and *in vivo* by downregulating the expression of cell cycle and energy metabolism genes in glioma stem-like cells (75).

Among these receptors, MT1 appears to play an important role in regulating stemness-associated programs and drug sensitivity. In brain tumor stem cell models, MT1 activation disrupts the interaction between the oncogenic transcription factor c-Myc and nestin. Under physiological conditions, these proteins require a SUMOylation-dependent association to sustain aggressive cellular programs. Melatonin disrupts this interaction, leading to c-Myc destabilization, inhibition of neurosphere formation, G2/M cell-cycle arrest, and increased sensitivity to cytotoxic agents such as paclitaxel (78).

Beyond its effects on stemness, melatonin also disrupts multiple adaptive survival mechanisms. In glioma-initiating cells, it induces apoptosis and autophagy-mediated cytotoxicity (79). Furthermore, combining melatonin with the histone deacetylase inhibitor vorinostat inhibits oligomerization of the transcription factor EB (TFEB), a master regulator of lysosomal biogenesis, thereby impairing metabolic adaptation and markedly reducing tumorsphere growth (80). Likewise, co-administration of melatonin with temozolomide increases methylation of the ABCG2/BCRP promoter, effectively reversing multidrug resistance phenotypes in GSCs (81).

More recently, melatonin has been shown to remodel the tumor microenvironment. Preconditioning mesenchymal stromal cells with melatonin delays GBM progression by reprogramming the tumor niche and suppressing molecular programs associated with cell migration and extracellular matrix remodeling (82). Rather than acting solely as a circadian hormone, melatonin integrates epigenetic, metabolic, and receptor-mediated signaling pathways to destabilize stem-like cellular states and enhance therapeutic vulnerability. This positions the melatonin signaling axis as a promising strategy for targeting the dynamic cellular landscape underlying GBM progression.

## 4 Underexplored hormonal axes in GSCs

### 4.1 Thyroid hormones

Thyroid hormones (THs), primarily thyroxine (T4) and its biologically active metabolite, triiodothyronine (T3), are essential regulators of CNS development, controlling the proliferation, maturation, and differentiation of glial lineages (83). In glioblastoma, however, thyroid hormone signaling extends beyond its classical endocrine role and integrates into local molecular circuits that influence tumor cell proliferation, differentiation, and phenotypic identity (84). The genomic effects of T3 are mediated by thyroid hormone receptors (TRs), ligand-activated nuclear transcription factors whose activity depends on intracellular hormone availability and interactions with transcriptional corepressors and epigenetic remodeling complexes. A central mechanism governing local thyroid hormone homeostasis is regulation of type 2 iodothyronine deiodinase (D2), the enzyme that converts T4 into bioactive T3. In this context, the RNA-binding protein Musashi-1 (MSI1) binds to the 3’ untranslated region (UTR) of DIO2 mRNA, repressing its translation and thereby reducing intracellular T3 availability. Disruption of the MSI1-D2-T3 axis alters thyroid hormone-dependent transcriptional programs that regulate glial proliferation and differentiation (85). These findings underscore the importance of local thyroid hormone homeostasis as a critical determinant of the balance between growth and differentiation within the CNS.

In the tumor setting, this regulatory network becomes even more complex. Glioma cells release exosomes containing signaling molecules that can reprogram neighboring neural stem cells (NSCs), diverting their normal differentiation toward a less differentiated phenotype through aberrant activation of the NOTCH and STAT3 pathways (86). This process is closely linked to remodeling of the tumor microRNA landscape. Among these alterations, miR-100 downregulation has emerged as a key event. Experimental restoration of miR-100, either by synthetic mimics *in vitro* or by tetracycline-inducible expression systems *in vivo*, significantly reduces tumorigenicity and decreases the Ki-67 proliferation index (87). Mechanistically, miR-100 promotes degradation of the nuclear corepressor SMRT/NCOR2, a transcriptional repressor that suppresses TRs’ activity in the absence of ligand. Loss of SMRT/NCOR2 consequently relieves TR-mediated transcription and reshapes epigenetic programs associated with cellular differentiation (87). This evidence indicates that thyroid hormone signaling, as part of an integrated network, does not operate as an isolated endocrine pathway but rather as an integrated network with epigenetic mechanisms, corepressor complexes, and microRNAs that dynamically regulate the balance between cell proliferation and differentiation-associated transcriptional programs. From this perspective, thyroid hormones may influence differentiation-associated cellular programs of glioblastoma.

### 4.2 Prolactin

In the CNS, prolactin (PRL) may act through local production and receptor signaling via PRLR. It exerts autocrine and paracrine effects on glial cells, regulating proliferation and modulating intracellular signaling, including cytosolic calcium dynamics (88). In GBM, both prolactin and its receptor (PRLR) are overexpressed relative to non-neoplastic brain tissue. Activation of this signaling axis induces STAT5 phosphorylation, promoting invasion and the molecular programs associated with an aggressive phenotype (89). The biological relevance of this pathway is supported by pharmacological inhibition studies. The recombinant PRLR antagonist ABD-PrlRA, a fusion protein engineered with an albumin-binding domain to prolong its biological half-life, effectively blocks STAT5 phosphorylation and suppresses the oncogenic potential of glioma cells *in vitro* (90).

Histopathological analyses of patient specimens and preclinical models have further demonstrated widespread co-expression of PRL and PRLR in glioblastoma tissues, supporting an intratumoral endocrine circuit with potential therapeutic relevance (91). In addition, PRL/PRLR signaling contributes to adaptive responses under therapeutic pressure. In human GBM cell lines and murine xenograft models, pharmacological inhibition of integrins induces compensatory activation of prolactin signaling, suggesting that this pathway participates in mechanisms that promote tumor survival during treatment (92).

Microenvironmental factors can also influence activation of the PRL/PRLR axis. Human cytomegalovirus (HCMV) infection, frequently detected in glioblastoma tissues, increases expression of both PRL and PRLR, integrating virus-induced signaling with endocrine pathways that support tumor growth (89–93). Although the specific role of prolactin signaling in glioma stem cells remains incompletely characterized, available data suggest that PRL/PRLR signaling may contribute to invasive behavior, survival, and adaptive stress signaling. Consequently, prolactin signaling may represent an additional endocrine modulator of cell-state plasticity, influencing the dynamic transitions between functional states that drive glioblastoma progression.

**Figure 1** shows the integrated hormonal modulation of GBM stemness-associated programs and adaptive cell state. Hormonal signals, including sex steroids, metabolic hormones, glucocorticoids, melatonin, thyroid hormones, and prolactin, converge on shared intracellular signaling pathways, including JAK/STAT, PI3K/AKT/mTOR, MAPK/ERK, and NF-κB, and **Table 1** summarizes the hormonal signaling pathways discussed in this review, including their primary receptors, reported biological effects in glioblastoma, evidence associated with stemness or stem-like phenotypes, interactions with therapeutic response or resistance, and their potential roles in cell-state regulation.

**Figure 1:**
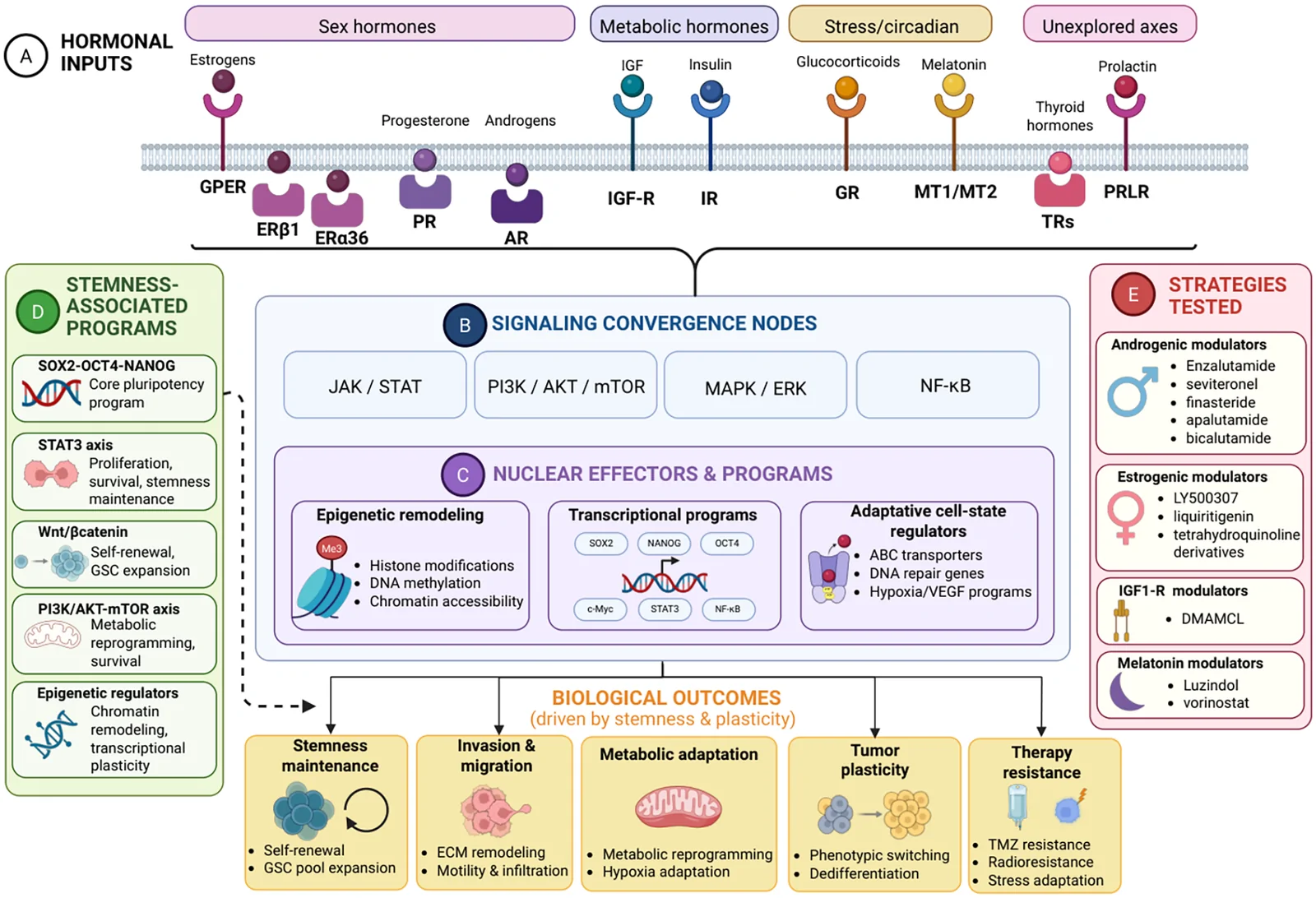
Integrated hormonal modulation of glioblastoma stemness-associated programs and adaptive cell states. Hormonal signals, including sex steroids, metabolic hormones, glucocorticoids, melatonin, thyroid hormones, and prolactin, converge on shared intracellular signaling pathways, including JAK/STAT, PI3K/AKT/mTOR, MAPK/ERK and NF-κB. Rather than acting as independent regulators, these signaling networks coordinate epigenetic and transcriptional programs that govern stem-like cellular states, including those controlled by SOX2, NANOG, and OCT4. Through these mechanisms, endocrine signaling may promote stemness maintenance, invasion, metabolic adaptation, therapeutic resistance, and ultimately tumor recurrence. The image summarizes the conceptual framework presented in this review.

**Table 1:** Hormone-related signaling pathways in glioblastoma: reported effects on stemness, therapeutic resistance, and cell-state regulation.

Hormonal axis	Main receptor/ context	GBM Reported effect	Stemness-associated evidence	Treatment resistance/Standard of Care interaction	Plasticity evidence	Key refs
Estrogens	ERα isoforms, ERβ isoforms, GPER; isoform-dependent	Context-dependent: ERβ1/ERβ activation can be suppressive; ERα36/ERβ5/GPER-related effects can support aggressive phenotypes in specific models.	ERβ agonism ↓ neurosphere formation and SOX2/CD133/SSEA-1 in patient-derived GSCs.	Preclinical evidence includes receptor modulation with effects on treatment sensitivity; clinical benefit not established.	Mostly indirect unless a study demonstrates state transition; marker/sphere changes alone = stemness-associated.	21–27
Progesterone	PR; concentration-dependent; PIBF-related local effects	Context-dependent: physiological-range exposure reported as pro-growth/invasive; high pharmacological doses can suppress glycolysis/induce senescence.	Gliosphere number/size increased in GBM cell-line-derived 3D models.	Combination with TMZ studied preclinically; effects depend on dose/context.	Indirect/associated; reversible state transitions not directly established in cited studies.	28–34
Androgens	AR; 5α-reductase-dependent androgen metabolism	Predominantly tumor-promoting AR signaling in reviewed GBM models; antagonism suppresses several malignant phenotypes.	AR inhibition ↓ CD133+ fraction, neurosphere formation, tumor-initiating frequency; ↓NANOG/OCT4/SOX2.	Enzalutamide/ seviteronel ↑radiosensitivity AR blockade sensitized GSC models to TMZ	Stem-state modulation supported; use “plasticity” cautiously unless transition/reversibility measured.	35–43
IGF/insulin	IGF-1R, IR; endocrine plus local/autocrine/paracrine context	Generally, supports survival/metabolic adaptation in the reviewed context.	IGF-1R linked to adaptive radioprotection and stem-like programs.	Strong preclinical link to radioresistance; miR-603/IGF1 pathway induced after radiation.	Adaptive state changes are supported in specific treatment contexts; not evidence of a universal endocrine network.	44–68 +
Glucocorticoids/DEX	GR; clinical exposure is pharmacological	DEX associated with adverse survival in meta-analysis; preclinical GR activation can promote stem-like/mesenchymal programs.	DEX ↑ neurosphere formation and CD133 in primary GBM cultures.	DEX induced TMZ resistance in primary cultures; clinically used for edema, so benefit–risk context is essential.	Transcriptomic reprogramming/mesenchymal programs provide stronger plasticity-related evidence than markers alone.	69–71
Melatonin	MT1, MT2; receptor effects can be opposing	Predominantly tumor-suppressive in reviewed GSC models.	↓tumorspheres, clonogenicity and SOX2/OCT4/NANOG/CD133; AKT–EZH2–STAT3 and EZH2–NOTCH1 implicated.	Reported chemosensitization and reversal of ABCG2/BCRP-associated multidrug resistance in preclinical models.	Evidence supports disruption of stem-like programs; specific claims of plasticity should follow the endpoints used.	72–79
Thyroid hormones	TRs; local D2/DIO2, MCT8/OATP1C1 transport, SMRT/NCOR2 context	Context-dependent; effects on proliferation/differentiation and local TH availability.	Direct GSC evidence remains limited in cited literature.	Direct interaction with standard GBM therapy not established in the cited section.	Evidence mainly concerns differentiation/ proliferation networks; direct GBM state-transition evidence limited.	80–84
Prolactin	PRLR/STAT5; systemic plus local autocrine/paracrine production	Predominantly pro-invasive/pro-survival in reviewed GBM models.	Direct evidence in purified GSCs is limited.	Compensatory PRL pathway activation reported after integrin-targeted therapy; SOC interaction not established.	Adaptive signaling is suggested, but direct reversible state transitions are not demonstrated.	85–90

↑, increases; ↓, decreases.

## 5 Interpreting sex differences in GBM: biological and non-biological determinants

Sex-based differences in GBM incidence and outcomes provide an important context for discussing hormonal signaling, but they do not constitute direct evidence of hormone dependence. Population-level patterns reflect the combined influence of cell-intrinsic sex biology, endocrine exposure, molecular tumor characteristics, and healthcare-related factors. Although epidemiological studies consistently report that glioblastoma occurs about 1.6-fold more often in men than in women (1), these sex differences should be interpreted within the broader context of healthcare disparities. Traditionally, this sexual dimorphism has been attributed to biological mechanisms, including gonadal steroid hormones and sex-specific genetic susceptibilities (94). However, recent evidence suggests that socioeconomic determinants and unequal access to healthcare may also substantially contribute to the incidence patterns observed across populations.

Socioeconomic disparities also appear to influence clinical outcomes and may modify sex-associated prognostic differences. In a population-based analysis of 230,601 patients with GBM, women had a slightly higher risk of death than men after adjusting for socioeconomic variables (Hazard Ratio [HR] = 1.07; 95% CI: 1.05–1.09) (95). These findings suggest that delayed diagnosis may attenuate -or even outweigh-, the biological advantages often attributed to female sex hormones. Likewise, a recent study showed that household income is a strong, independent predictor of survival in patients with IDH-wildtype GBM; in particular, lower household income predicted poorer overall survival (96).

These observations highlight that the endocrine biology of GBM cannot be understood in isolation from the social and healthcare environments where the disease is diagnosed and treated. Sex-specific differences in incidence and outcomes therefore likely arise from the complex interplay among endocrine regulation, genetic susceptibility, and structural determinants of health that shape access to diagnosis, treatment, and clinical follow-up. Recognizing these sources of epidemiological bias is essential to avoid attributing population-level differences exclusively to hormonal mechanisms and to enable a more integrated interpretation of glioblastoma within its clinical and social context.

## 6 Conclusions

The evidence reviewed here supports a conceptual shift in how GBM is understood. Rather than a disease driven exclusively by genomic alterations or isolated hormonal pathways, GBM may be viewed as an endocrine-responsive malignancy in which multiple hormonal signals can modulate tumor cell states, stem-like phenotypes, and therapeutic adaptation. This conceptual framework should not be interpreted as defining endocrine regulation as a hallmark of GBM; instead, it offers a testable model that integrates systemic and local hormonal influences with the molecular heterogeneity of the disease.

From this perspective, sex hormones, glucocorticoids, the IGF/insulin axis, melatonin, thyroid hormones, and prolactin no longer represent independent signaling pathways but instead form a highly interconnected regulatory network. Although these endocrine axes respond to distinct physiological stimuli, they converge on a limited set of conserved molecular nodes, including PI3K/AKT/mTOR, MAPK/ERK, JAK/STAT3, EZH2, and other epigenetic regulators that integrate endocrine, metabolic, and microenvironmental information. The coordinated activation of these signaling networks ultimately governs programs of stemness, adaptive reprogramming, therapeutic response, and tumor evolution.

Accordingly, we propose that the primary role of endocrine signaling in GBM is not solely to promote or suppress tumor growth, but to regulate the stability and transitions among the cellular states that constitute the tumor ecosystem. Hormonal signals integrate systemic physiological cues with microenvironmental stimuli, thereby facilitating or restricting transitions between proliferative, quiescent, invasive, and stem-like phenotypes. Under this model, cell-state plasticity may emerge as a system-level property resulting from the continuous integration of endocrine, metabolic, immune, and environmental signals rather than from individual genetic alterations alone.

It is equally important to recognize that the historical tendency to study these signaling pathways in isolation reflects not a limitation of the field but a consequence of the reductionist context that has traditionally guided biomedical research. Although this approach has been essential for establishing causal mechanisms, it has also promoted a fragmented view of GBM biology that underestimates the extensive crosstalk among endocrine, molecular, metabolic, and microenvironmental regulators. Such a framework will be essential for developing therapeutic strategies that more accurately reflect the biological and clinical complexity of glioblastoma.

## 7 Future directions

To translate these concepts into clinical practice and overcome the persistent therapeutic limitations in glioblastoma, several research priorities may be considered.

1. Understanding the convergence of endocrine signals. Future studies should move beyond investigations of individual hormonal pathways to elucidate how multiple endocrine signals exert context-dependent effects, ranging from neuroprotection to the promotion of stemness, depending on the epigenetic context and functional state of tumor cells. Understanding how sex hormones, glucocorticoids, insulin, and other endocrine mediators coordinately regulate metabolic adaptation and cell-state plasticity may reveal shared regulatory nodes that sustain therapeutic resistance and represent attractive targets for combinatorial interventions.
2. Re-evaluating the clinical use of dexamethasone. Although dexamethasone remains indispensable for controlling peritumoral edema and alleviating neurological symptoms, accumulating evidence suggests that prolonged exposure may inadvertently promote stemness-associated programs, mesenchymal transition, and therapeutic resistance. Therefore, optimizing the balance between its clinical benefits and potential protumorigenic effects should be a priority in future translational and clinical studies.
3. Integrating chronobiology into precision neuro-oncology. Chronotherapy, which adjusts treatment timing with circadian rhythms and fluctuations in endocrine signaling, is a promising strategy to enhance therapeutic efficacy while minimizing systemic toxicity. Despite a compelling biological rationale, this field remains largely unexplored in glioblastoma and warrants systematic clinical investigation.
